# Extraction and identification of synovial tissue-derived exosomes by different separation techniques

**DOI:** 10.1186/s13018-020-01604-x

**Published:** 2020-03-09

**Authors:** Pu Chen, Anmin Ruan, Jun Zhou, Liuwei Huang, Xiaozhe Zhang, Yufeng Ma, Qingfu Wang

**Affiliations:** 1 Department of Orthopaedic Surgery, Beijing University of Chinese Medicine Third Affiliated Hospital, No. 51, Xiaoguan Street, Anding Gate, Chaoyang District, Beijing, China; 2 Southern Medical University, Guangzhou, Guangdong Province China

**Keywords:** Exosomes, Extracellular vesicles, Osteoarthritis, Synovial tissues

## Abstract

**Objective:**

The aim of this study is to compare the efficiency of different separation techniques for extracting synovial tissue-derived exosomes.

**Methods:**

The synovial tissue discarded during knee arthroscopy or total knee arthroplasty surgery was collected from the Third Affiliated Hospital of Beijing University of Chinese Medicine. Ultracentrifugation (UC), filtration combined with size exclusion chromatography (SECF), and 8% polyethylene glycol (PEG) were used to extract synovial tissue-derived exosomes. Transmission electron microscopy (TEM), nanoparticle tracer analysis (NTA), and Western Blot (WB) were used to detect the morphology, particle size, and biomarker proteins (CD9, CD63, Flotillin-1, and calnexin) of exosomes.

**Results:**

The extracts of enriched round and discoid vesicles were successfully extracted with UC, SECF, and PEG. The results of TEM have shown that all three extraction methods can extract circular or elliptical vesicles with disc- and cup-shaped structures from the synovial tissue, with the diameter is about 30–150 nm. NTA suggested the main peaks of three groups of exosomes are around 100–120 nm, and the concentration of the three groups of exosomes was greater than 1 × 10^10^/ml. The results of WB showed that three positive protein markers (CD9, CD63, and Flotillin-1) were highly expressed in the suspension extracted by the three methods and low in the synovial tissue. However, the negative protein (calnexin) was highly expressed in synovial tissues and PEG group, while low in UC and SECF group.

**Conclusion:**

Morphology, particle size, and labeled protein marker detection confirmed that UC, SECF, and PEG can extract exosomes derived from synovial tissue; UC and SECF are more recommended for the extraction of synovial tissue-derived exosomes, which provides a methodological basis for studying the function and mechanism of synovial tissue exosomes in the future.

## Introduction

Osteoarthritis (OA) is a musculoskeletal disease characterized by synovial inflammation, progressive cartilage wear, and subchondral bone hyperplasia [1, 2]. It was reported that OA is one of the top ten causes of lower limb disability [3, 4]. For many years, cartilage damage has been considered to be the main pathogenesis of OA. However, with the deepening of research, the important role of soft tissues around the joints such as synovial tissues and fat pad in the pathogenesis of OA has been recognized more and more [2, 5–7]. Recent studies have pointed out that synovial inflammation is a precursor to radiological OA [8], has a significant positive correlation with OA symptoms [9], and further aggravates cartilage damage [10]. In the previous study, we investigated the relationship between synovial membrane, articular cartilage, and the innate immune system in different degrees of KOA rats during pathogenesis, and found that the innate immune system is associated with synovial membrane and articular cartilage at all stages of the disease. It also suggests that the synovial membrane may affect the pathological changes of articular cartilage through the innate immune system, thus affecting the progression of OA [11]. However, how synovial tissue affects cartilage, how the synovial inflammatory transmitted between cells and tissues remains to be studied.

Exosomes are 30–150 nm-sized extracellular vesicles, with lipid bilayer membranes, and released by many different types of cells [12]. As reported, exosomes widely existed in various cell supernatants and biological fluids [13, 14] such as plasma, serum, cerebrospinal fluid, urine, and milk. They contain many biologically active substances secreted by recipient cells, such as non-cording RNA (ncRNA), proteins, and mRNA. And it plays an important role in the extracellular microenvironment and intercellular communication [12, 15–18]. Kato [19] extracted exosomes from the supernatant of IL-1β-induced synovial fibroblasts and then added exosomes to chondrocytes. The results showed that the matrix metalloproteinase-13 (MMP-13) was highly upregulated and the type II collagen was significant downregulated, which accelerated the progression of OA. This suggests that synovial tissue may regulate chondrocytes by secreting exosomes. Number of articles [15–22] has conducted detailed studies on how to extract cell supernatants and exosomes in body fluids, but few of articles had studied the extraction and identification of exosomes in tissues. To the best of our knowledge, any article has been published on how to extract and identify exosomes from synovial tissue. Therefore, the purpose of this study was to extract exosomes from synovial tissue by different separation techniques, and then identify and compare the extracted exosomes by transmission electron microscopy (TEM), nanoparticle tracer analysis (NTA), and Western Blot (WB).

## Methods

### Main instruments and reagents

Exosome-free fetal bovine serum was purchased from ABW (Uruguay); Dulbecco’s Modified Eagle Medium high-glucose (DMEM-H) was purchased from Hyclone (Boston, USA); antibiotic mixture (penicillin and streptomycin) was obtained from Invitrogen (CA, USA); 0.22 μm filter was purchased from Millipore (USA); polyethylene glycol (PEG) 6000 was obtained from Sigma (USA); BCA protein concentration assay kit, constant temperature incubator, and cryostat centrifuge were purchased from Thermo Scientific (USA); size exclusion chromatography and filtration kit were purchased from Echo Biotech (Beijing, China); primary antibodies against CD9, CD63, Flotillin-1, and calnexin were purchased from CST (Cambridge, MA, USA); goat anti-rabbit and goat anti-mouse horseradish peroxidase conjugates were purchased from Bio-Rad Laboratories (Proteintech, USA); inverted phase contrast microscope was obtained from Olympus (Japan); nanoparticle tracking analyzer was obtained from PMX (Germany); transmission electron microscope H-7650 was obtained from Hitachi (Japan).

### Collection and treatment of synovial tissue specimens

The study was approved by the Third Affiliated Hospital of Beijing University of Chinese Medicine and its ethics committee. De-identified discarded human synovial tissue samples were used for this study. From May 2019 to July 2019, a total of 18 samples of synovial tissue were obtained from the discarded synovial tissue of knee arthroscopy or joint replacement surgery in the Third Affiliated Hospital of Beijing University of Chinese Medicine. All patients underwent arthroscopic or joint replacement surgery for primary knee osteoarthritis. Synovial tissue was randomly divided into 3 groups, polyethylene glycol (PEG) group, ultracentrifugation (UC) group, and size exclusion chromatography and filtration (SECF) group, with 6 cases in each group. The synovial tissue obtained during the operation was placed in PBS containing 1% antibiotic mixture, placed in an ice box, and quickly transferred to a clean bench. Synovial tissue was immediately placed in a new culture dish; PBS containing 1% antibiotic mixture was added thereto, washed 3 times for 5 min each time. The cleaned synovial tissue was transferred to a new culture dish (60 mm), cut into pieces of 1 × 1 × 1 mm^3^ by ophthalmic scissors, added 5-ml culture medium (containing 10% exosome-free fetal bovine serum and 1% antibiotic mixture), and then placed in constant temperature incubator with 37 °C, 5% CO_2_. After 24 h, the culture supernatant was collected and the medium was changed, and the supernatant was collected again 24 h later. The twice collected supernatant was filtered through a 0.22-μm filter and then placed in − 80 °C for storage.

### Extract exosomes from collected culture supernatant

For the PEG group, an equal volume of 16% PEG 6000 solution (with a final solubility of 8% PEG 6000) was added to the collected medium and overnight at 4 °C. The mixture was centrifuged at 12,000×*g* for 30 min at 4 °C in the following day, the supernatant was discarded and the pellet was resuspended by PBS. The suspension was centrifuged at 12,000×*g* for 30 min again, resuspended by PBS, and then placed in − 80 °C for storage.

For the UC group, the collected medium was centrifuged at 2000×*g* for 20 min to eliminate the cells and then centrifuged at 10,000×*g* for 20 min to eliminate the cell debris, followed by ultracentrifugation at 150,000×*g* for 120 min. The supernatant was discarded, resuspended by PBS, and then placed in − 80 °C for storage.

For the SECF group, all procedures were performed in strict accordance with the kit instructions.

### Transmission electron microscopy (TEM)

To detect the morphology of exosomes, transmission electron microscopy was performed. Take 10 μl exosome suspensions on copper grid, after incubating for 10 min at room temperature, rinse with sterile distilled water and absorb excess liquid from absorbent paper. Then, 10 μl of 2% uranyl acetate was pipetted onto the copper grid for 1 min, and the filter paper was sucked off and dried under an incandescent lamp for 2 min. The copper mesh was observed under transmission electron microscopy and imaged at 80 kV.

### Nanoparticle tracer analysis (NTA)

Nanoparticle tracking analysis was performed to determine particle size and concentration of exosomes. Exosome suspensions were appropriately diluted using PBS buffer (with a final concentration of 1 × 10^7^–10^8^/ml) to measure the particle size and concentration. The suspension is irradiated with a laser light source, and the scattered light of the nanoparticles is detected. The nanoparticle concentration is calculated by counting the number of scattering particles.

### Western Blot (WB)

As described in the previous article [11], after quantified by the BCA protein, each group of exosomes was added to each well and electrophoresed. Subsequently, the protein was transferred to a PVDF membrane, and the 5% skim milk powder was incubated for 1 h at room temperature, then incubated with primary antibody (4 °C overnight or room temperature for 2 h), and TBST was washed 4 times for 5 min each time. Subsequently, the mixture with second antibody was incubated for 1 h at room temperature, and again washed with TBST for 5 min × 8 times, and ECL was added for development.

### Statistical analyses

Data obtained from this research were presented as the mean ± standard deviation (SD). All analyses were performed using the GraphPad Prism 7.0 software. The differences between different groups were compared using *t* test or one-way ANOVA. *P* < 0.05 was considered statistically significant.

## Results

### Morphological characteristics of synovial tissue-derived exosomes

**Fig. 1:**
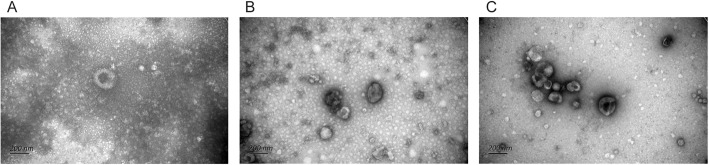
Morphological characteristics of synovial tissue-derived exosomes extracted by three different extraction methods. **a** The PEG group. **b** The UC group. **c** The SECF group

Transmission electron microscopy (TEM) was used to observe the morphology of synovial tissue-derived exosomes. As presented in Fig. 1, all three extraction methods can extract circular or elliptical vesicles with disc- and cup-shaped structures from the synovial tissue, with the diameter is about 30–150 nm, which is consistent with the morphology feature of exosomes. In terms of the background of TEM, the background of PEG group is more blurred and more impurities, while the SECF group is the cleanest, with the least impurities and clearer morphology of exosomes.

### Size distribution and concentration of synovial tissue-derived exosomes

**Fig. 2:**
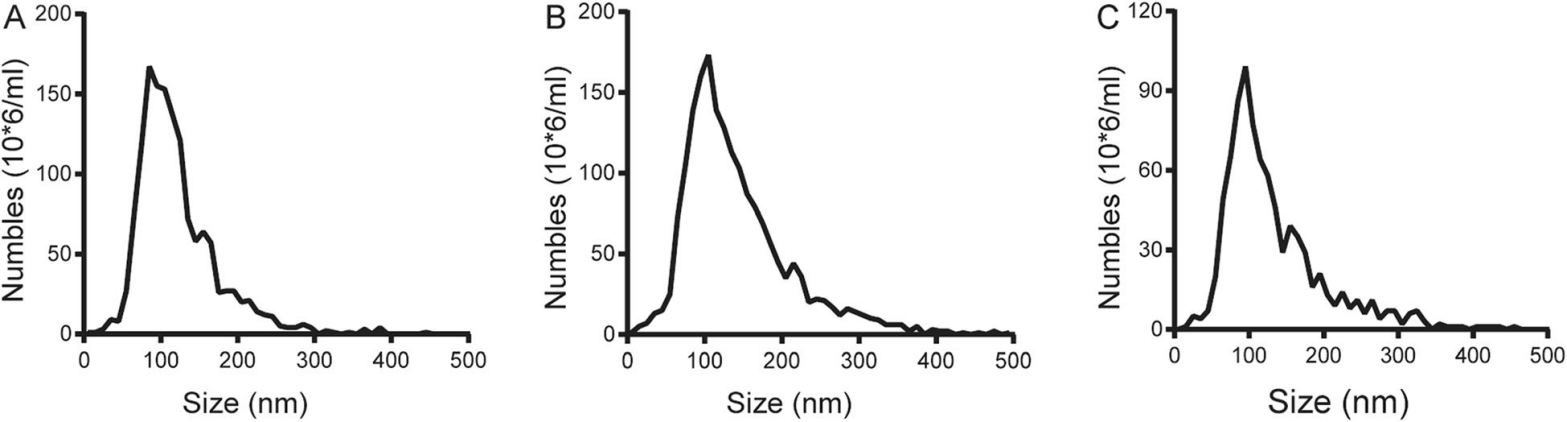
Morphological characteristics of synovial tissue-derived exosomes extracted by three different extraction methods. **a** The PEG group. **b** The UC group. **c** The SECF group

The particle size and concentration of synovial tissue-derived exosomes were assessed by nanoparticle tracking analysis (NTA). As shown in Fig. 2, the main peaks of three groups of exosomes are around 100–120 nm. The concentration of the three groups of exosomes was greater than 1 × 10^10^/ml. The particle size distribution and the cumulative percentage of the interval were presented in Table 1.

**Table 1:** The particle size distribution and the cumulative percentage of the interval

Size (nm)	PEG			UC			SECF		
	Number (10^6^/ml)	Percent (%)	Cumulative percent (%)	Number (10^6^/ml)	Percent (%)	Cumulative percent (%)	Number (10^6^/ml)	Percent (%)	Cumulative percent (%)
5	1	0.07	0.07	1.00	0.05	0.05	0.00	0.00	0.00
15	1	0.07	0.14	5.00	0.27	0.33	1.00	0.12	0.12
25	3	0.21	0.35	7.00	0.38	0.71	5.00	0.58	0.69
35	9	0.64	0.99	13.00	0.71	1.41	4.00	0.46	1.15
45	8	0.56	1.55	15.00	0.81	2.23	7.00	0.81	1.96
55	27	1.91	3.46	25.00	1.36	3.59	20.00	2.30	4.26
65	73	5.15	8.61	73.00	3.97	7.55	49.00	5.65	9.91
75	118	8.33	16.94	105.00	5.70	13.25	65.00	7.49	17.40
85	167	11.79	28.72	139.00	7.55	20.80	86.00	9.91	27.30
95	155	10.94	39.66	160.00	8.69	29.49	99.00	11.41	38.71
105	153	10.80	50.46	173.00	9.40	38.89	77.00	8.87	47.58
115	137	9.67	60.13	139.00	7.55	46.44	64.00	7.37	54.95
125	121	8.54	68.67	128.00	6.95	53.39	58.00	6.68	61.64
135	72	5.08	73.75	113.00	6.14	59.53	46.00	5.30	66.94
145	58	4.09	77.84	103.00	5.59	65.13	29.00	3.34	70.28
155	64	4.52	82.36	87.00	4.73	69.85	39.00	4.49	74.77
165	57	4.02	86.38	79.00	4.29	74.14	35.00	4.03	78.80
175	26	1.83	88.21	69.00	3.75	77.89	29.00	3.34	82.14
185	27	1.91	90.12	57.00	3.10	80.99	16.00	1.84	83.99
195	27	1.91	92.03	45.00	2.44	83.43	21.00	2.42	86.41
205	20	1.41	93.44	35.00	1.90	85.33	13.00	1.50	87.90
215	21	1.48	94.92	44.00	2.39	87.72	9.00	1.04	88.94
225	14	0.99	95.91	36.00	1.96	89.68	14.00	1.61	90.55
235	12	0.85	96.75	20.00	1.09	90.77	8.00	0.92	91.47
245	11	0.78	97.53	22.00	1.20	91.96	11.00	1.27	92.74
255	5	0.35	97.88	21.00	1.14	93.10	6.00	0.69	93.43
265	4	0.28	98.17	17.00	0.92	94.02	11.00	1.27	94.70
275	4	0.28	98.45	12.00	0.65	94.68	4.00	0.46	95.16
285	6	0.42	98.87	16.00	0.87	95.55	7.00	0.81	95.97
295	4	0.28	99.15	14.00	0.76	96.31	7.00	0.81	96.77
305	0	0.00	99.15	12.00	0.65	96.96	2.00	0.23	97.00
315	2	0.14	99.29	10.00	0.54	97.50	6.00	0.69	97.70
325	1	0.07	99.36	9.00	0.49	97.99	7.00	0.81	98.50
335	0	0.00	99.36	6.00	0.33	98.32	3.00	0.35	98.85
345	1	0.07	99.44	6.00	0.33	98.64	0.00	0.00	98.85
355	0	0.00	99.44	6.00	0.33	98.97	2.00	0.23	99.08
365	3	0.21	99.65	2.00	0.11	99.08	1.00	0.12	99.19
375	0	0.00	99.65	5.00	0.27	99.35	1.00	0.12	99.31
385	4	0.28	99.93	0.00	0.00	99.35	1.00	0.12	99.42
395	0	0.00	99.93	3.00	0.16	99.51	0.00	0.00	99.42
405	0	0.00	99.93	2.00	0.11	99.62	1.00	0.12	99.54
415	0	0.00	99.93	2.00	0.11	99.73	1.00	0.12	99.65
425	0	0.00	99.93	0.00	0.00	99.73	1.00	0.12	99.77
435	0	0.00	99.93	1.00	0.05	99.78	1.00	0.12	99.88
445	1	0.07	100.00	0.00	0.00	99.78	0.00	0.00	99.88
455	0	0.00	100.00	1.00	0.05	99.84	1.00	0.12	100.00
465	0	0.00	100.00	0.00	0.00	99.84	0.00	0.00	100.00
475	0	0.00	100.00	2.00	0.11	99.95	0.00	0.00	100.00
485	0	0.00	100.00	0.00	0.00	99.95	0.00	0.00	100.00
495	0	0.00	100.00	1.00	0.05	100.00	0.00	0.00	100.00

### Determination of the protein concentration of synovial tissue-derived exosomes with BCA

**Fig. 3:**
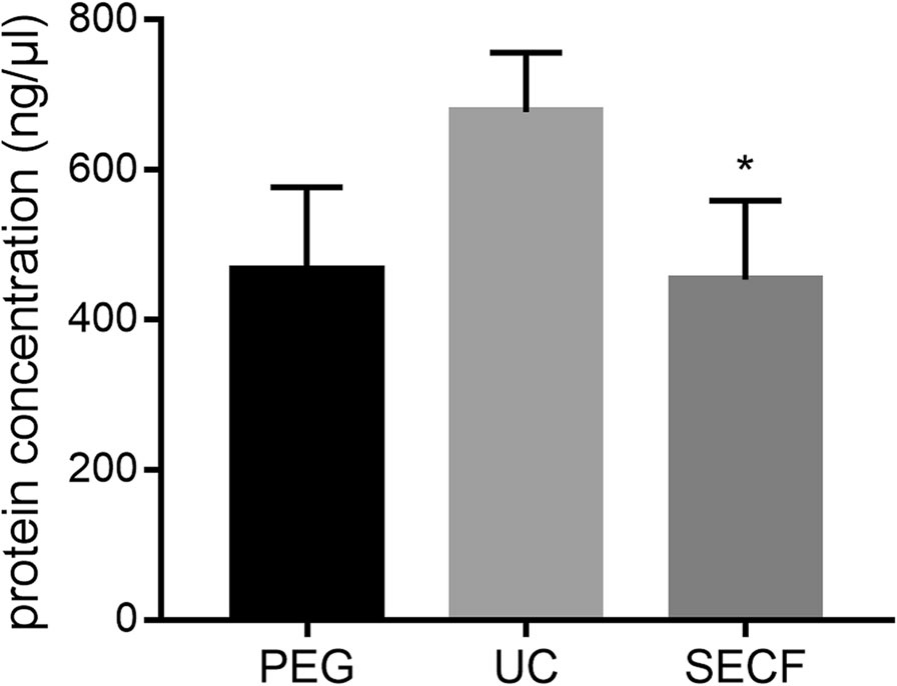
The protein concentration of synovial tissue-derived exosomes. *, compared with the UC group, *P* < 0.05

The concentration of protein in the three groups of synovial tissue-derived exosomes was measured by BCA assay, measured the absorbance at OD562nm, drawn the standard curve. The protein concentration was determined according to the standard curve equation. As shown in Fig. 3, the UC group had the highest protein concentration, and the SECF group was significantly different from the UC group (*P* < 0.05). No significant difference was found between the PEG group and the other two groups.

### The results of exosome biomarker tested by Western Blot

**Fig. 4:**
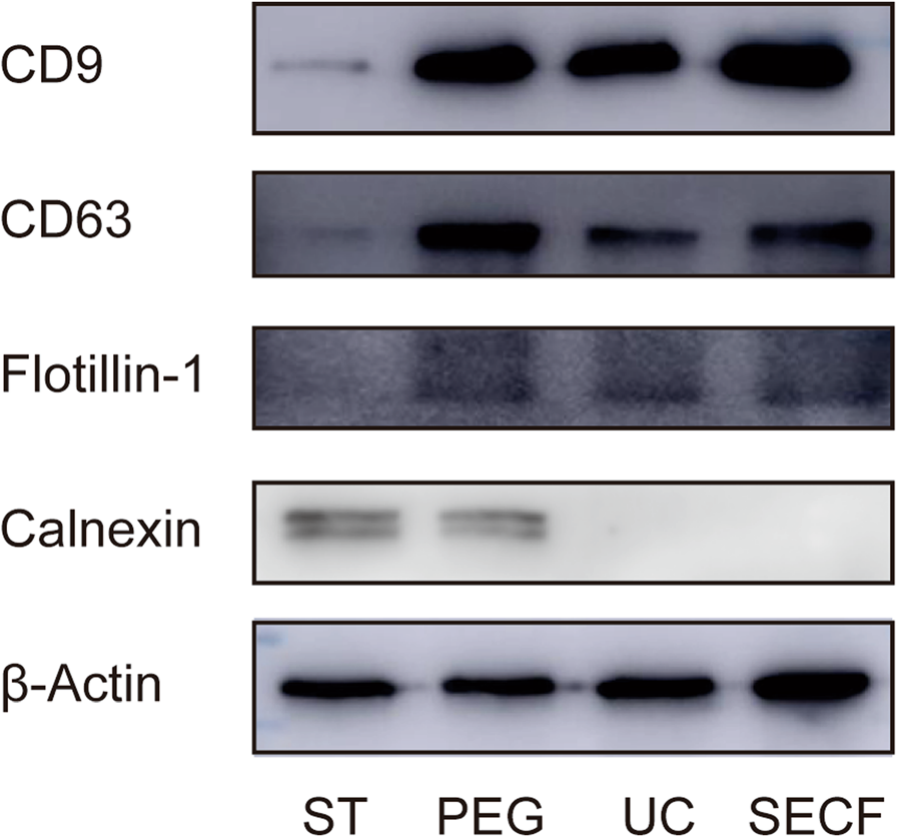
The exosomes biomarker detection results with WB. ST synovial tissues

The exosome biomarker proteins (CD9, CD63, Flotillin-1, and calnexin) were detected by WB. The results showed that CD9, CD63, and Flotillin-1 were highly expressed in the suspension extracted by the three methods and low in the synovial tissue. However, calnexin was highly expressed in the synovial tissue and PEG group, while low in the UC and SECF group (Fig. 4).

## Discussion

Since mRNA and microRNA were discovered in extracellular vesicles in 2007, the heat of extracellular vesicles has increased [23]. In particular, after the research on extracellular vesicles award the Nobel Prize in Physiology or Medicine in 2013, the research on the regulation mechanism of cell vesicle transport was a spurt-like growth. Vesicles can be divided into three categories according to origin, biological function, and transformation outcome: apoptotic bodies (1–4 μm), microvesicles (0.1–1 μm), and exosomes, which are the focus of scholars [24]. As exosomes were initially found in reticulocytes, exosomes were found in cell supernatants such as macrophages, T lymphocytes, epithelial cells, and nerve cells. Exosomes are also found in blood, semen, ascites, milk, etc. It is currently believed that exosomes are found in almost all mammalian cells and in the body. Exosomes are extremely rich in content. According to statistics, there are currently 9769 proteins, 3408 mRNAs, 1116 lipids, and 2838 microRNAs found in exosomes (www.exocarta.org). These data are mainly derived from early research results, the latest research results have not been included in time and the database was last updated on September 12, 2016. The donor cells secrete exosomes, envelop the target protein, mRNA, and non-cording RNA, received by the recipient cells, and then exerting their intercellular communication and regulatory functions.

With the deepening of exosome research, how to isolate and identify exosomes is one of the important methodological foundations for continued research. At present, a large amount of literature has been published on the extraction of exosomes from cell supernatants and body fluids, such as ultracentrifugation, sucrose density gradient centrifugation, immunomagnetic beads, filtration, ultrafiltration, and various commercial kits. However, few literatures had studied how to extract exosomes from tissues. Lin [25] extracted exosomes from kidney tissue, digested in the kidney, with collagenase and trypsin for 120 min at 37 °C then took the supernatant for ultracentrifugation; detected the exosomes with TEM, NTA, and the exosome biomarker proteins (CD9, CD63, and Alix); and then identified as exosomes. Deng [26] extracted exosomes from adipose tissue. After washing with PBS, the adipose tissue was cut into pieces smaller than 4 mm, transferred to a 6-well plate, and added to a DMEM solution containing 1% antibiotic mixture and 10% FBS. The supernatant was separated and the exosomes were separated by gradient centrifugation. Beth [27] isolated the extracellular vesicles in the placental tissue, chopped the placenta tissue, placed in the medium, and then placed in a constant temperature incubator for 24 h. The supernatant was collected, and separated the vesicles by ultracentrifugation. Lucia [28] has published a protocol on how to extract exosomes from cell lines and tissues explants, which we have used in our research.

Synovial inflammation, as one of the typical pathological features of OA, plays an important role in the development of OA [2, 5–7, 11]. However, as of now, any article has been published on how to extract and identify exosomes in synovial tissue. Importantly, more and more researchers consider the synovial membrane and intra-articular fat pad as an anatomical unit in OA pathogenesis and pain, and inflammation of synovial membrane in the knee joint may play a central role in OA and affect not only cartilage but also intra-articular fat pad [7, 29, 30]. Moreover, previous team studies have shown that synovial inflammation may affect articular cartilage damage through the innate immune system, aggravating the course of OA [11]. Therefore, how to separate and extract exosomes from synovial tissue is important for studying the function and mechanism of synovial tissue exosomes.

Although there are various methods for extracting exosomes, each method has its advantages and disadvantages. In 2018, the International Society for Extracellular Vesicles published a guide to extracellular studies [12]. In this article, the International Society for Extracellular Vesicles summarizes the methods of vesicle extraction and classifies them into three categories based on the results of the separation: high recovery and low specificity, intermediate recovery and intermediate specificity, and low recovery and high specificity. We selected one method from each of these three categories to extract synovial tissue exosomes, which were PEG, UC, and SECF. In addition, we selected three positive protein markers (included two transmembrane proteins and one cytosolic protein, CD9, CD63, and Flotillin-1) and one negative protein marker (calnexin) for exosomes indicating markers. The result showed that the positive protein markers were highly expressed in the exosomes and low in synovial tissues, while the negative protein was highly expressed in synovial tissues and PEG group and low in UC and SECF group. This phenomenon may be explained by the fact that PEG can enrich a large amount of impurity proteins, which is also consistent with the recommendations in the guidelines [12], suggesting high concentration and low purity. At the same time, we used TEM and NTA to detect the morphology, particle size, and concentration of exosomes; the results were consistent with the characteristics of exosomes. Therefore, all three methods can extract exosomes from synovial tissue, while PEG group may be doped with a large amount of impurity proteins.

Taken together, UC and SECF are more recommended for the extraction of synovial tissue-derived exosomes, which lays a methodological basis for the subsequent study of the function and mechanism of synovial tissue-derived exosomes.

## Data Availability

All data are fully available without restriction.

## Abbreviations

NTA: Nanoparticle tracer analysis
OA: Osteoarthritis
PEG: Polyethylene glycol
SECF: Filtration combined with size exclusion chromatography
TEM: Transmission electron microscopy
UC: Ultracentrifugation
WB: Western Blot

## References

[1] Malemud CJ (2015). Biologic basis of osteoarthritis: state of the evidence. Curr Opin Rheumatol.

[2] Berenbaum F (2013). Osteoarthritis as an inflammatory disease (osteoarthritis is not osteoarthrosis!). Osteoarthritis Cartilage.

[3] Neogi T (2013). The epidemiology and impact of pain in osteoarthritis. Osteoarthritis Cartilage.

[4] Chen P, Huang L, Ma Y, Zhang D, Zhang X, Zhou J, Ruan A, Wang Q (2019). Intra-articular platelet-rich plasma injection for knee osteoarthritis: a summary of meta-analyses. J Orthop Surg Res.

[5] Mancarella L, Addimanda O, Cavallari C, Meliconi R (2016). Synovial inflammation drives structural damage in hand osteoarthritis: a narrative literature review. Curr Rheumatol Rev.

[6] Ozeki N, Muneta T, Koga H, Nakagawa Y, Mizuno M, Tsuji K, Mabuchi Y, Akazawa C, Kobayashi E, Matsumoto K (2016). Not single but periodic injections of synovial mesenchymal stem cells maintain viable cells in knees and inhibit osteoarthritis progression in rats. Osteoarthritis Cartilage.

[7] Belluzzi E, Stocco E, Pozzuoli A, Granzotto M, Porzionato A, Vettor R, De Caro R, Ruggieri P, Ramonda R, Rossato M (2019). Contribution of infrapatellar fat pad and synovial membrane to knee osteoarthritis pain. #N/A 2019.

[8] Atukorala I, Kwoh CK, Guermazi A, Roemer FW, Boudreau RM, Hannon MJ, Hunter DJ (2016). Synovitis in knee osteoarthritis: a precursor of disease?. Ann Rheum Dis.

[9] Guermazi A, Hayashi D, Roemer FW, Zhu Y, Niu J, Crema MD, Javaid MK, Marra MD, Lynch JA, El-Khoury GY (2014). Synovitis in knee osteoarthritis assessed by contrast-enhanced magnetic resonance imaging (MRI) is associated with radiographic tibiofemoral osteoarthritis and MRI-detected widespread cartilage damage: the MOST study. J Rheumatol.

[10] Roemer FW, Guermazi A, Felson DT, Niu J, Nevitt MC, Crema MD, Lynch JA, Lewis CE, Torner J, Zhang Y (2011). Presence of MRI-detected joint effusion and synovitis increases the risk of cartilage loss in knees without osteoarthritis at 30-month follow-up: the MOST study. Ann Rheum Dis.

[11] Wang H, Wang Q, Yang M, Yang L, Wang W, Ding H, Zhang D, Xu J, Tang X, Ding H (2018). Histomorphology and innate immunity during the progression of osteoarthritis: does synovitis affect cartilage degradation?. J Cell Physiol.

[12] Thery C, Witwer KW, Aikawa E, Alcaraz MJ, Anderson JD, Andriantsitohaina R, Antoniou A, Arab T, Archer F, Atkin-Smith GK (2018). Minimal information for studies of extracellular vesicles 2018 (MISEV2018): a position statement of the International Society for Extracellular Vesicles and update of the MISEV2014 guidelines. J Extracell Vesicles.

[13] Foers AD, Chatfield S, Dagley LF, Scicluna BJ, Webb AI, Cheng L, Hill AF, Wicks IP, Pang KC (2018). Enrichment of extracellular vesicles from human synovial fluid using size exclusion chromatography. J Extracell Vesicles.

[14] Johnsen KB, Gudbergsson JM, Skov MN, Pilgaard L, Moos T, Duroux M (2014). A comprehensive overview of exosomes as drug delivery vehicles—endogenous nanocarriers for targeted cancer therapy. Biochim Biophys Acta.

[15] Li D, Liu J, Guo B, Liang C, Dang L, Lu C, He X, Cheung HY, Xu L, Lu C (2016). Osteoclast-derived exosomal miR-214-3p inhibits osteoblastic bone formation. Nat Commun.

[16] Yang DW, Qian GB, Jiang MJ, Wang P, Wang KZ. Inhibition of microRNA-495 suppresses chondrocyte apoptosis through activation of the NF-kappaB signaling pathway by regulating CCL4 in osteoarthritis. Gene Ther. 2019. 10.1038/s41434-019-0068-5 30940879

[17] He S, Li Z, Yu Y, Zeng Q, Cheng Y, Ji W, Xia W, Lu S. Exosomal miR-499a-5p promotes cell proliferation, migration and EMT via mTOR signaling pathway in lung adenocarcinoma. Exp Cell Res. 2019. 10.1016/j.yexcr.2019.03.035 30978341

[18] Wu J, Kuang L, Chen C, Yang J, Zeng WN, Li T, Chen H, Huang S, Fu Z, Li J (2019). miR-100-5p-abundant exosomes derived from infrapatellar fat pad MSCs protect articular cartilage and ameliorate gait abnormalities via inhibition of mTOR in osteoarthritis. Biomaterials.

[19] Kato T, Miyaki S, Ishitobi H, Nakamura Y, Nakasa T, Lotz MK, Ochi M (2014). Exosomes from IL-1beta stimulated synovial fibroblasts induce osteoarthritic changes in articular chondrocytes. Arthrit Res Ther.

[20] Wang L, Wang C, Jia X, Yu J (2018). Circulating exosomal miR-17 inhibits the induction of regulatory T Cells via suppressing TGFBR II expression in rheumatoid arthritis. Cell Physiol Biochem.

[21] Sun H, Hu S, Zhang Z, Lun J, Liao W, Zhang Z (2019). Expression of exosomal microRNAs during chondrogenic differentiation of human bone mesenchymal stem cells. J Cell Biochem.

[22] Burke J, Kolhe R, Hunter M, Isales C, Hamrick M, Fulzele S (2016). Stem cell-derived exosomes: a potential alternative therapeutic agent in orthopaedics. Stem Cells Int.

[23] Valadi H, Ekstrom K, Bossios A, Sjostrand M, Lee JJ, Lotvall JO (2007). Exosome-mediated transfer of mRNAs and microRNAs is a novel mechanism of genetic exchange between cells. Nat Cell Biol.

[24] Lemoinne S, Thabut D, Housset C, Moreau R, Valla D, Boulanger CM, Rautou PE (2014). The emerging roles of microvesicles in liver diseases. Nat Rev Gastroenterol Hepatol.

[25] Lv LL, Feng Y, Wen Y, Wu WJ, Ni HF, Li ZL, Zhou LT, Wang B, Zhang JD, Crowley SD (2018). Exosomal CCL2 from tubular epithelial cells is critical for albumin-induced tubulointerstitial inflammation. J Am Soc Nephrol.

[26] Deng ZB, Poliakov A, Hardy RW, Clements R, Liu C, Liu Y, Wang J, Xiang X, Zhang S, Zhuang X (2009). Adipose tissue exosome-like vesicles mediate activation of macrophage-induced insulin resistance. Diabetes.

[27] Holder BS, Tower CL, Forbes K, Mulla MJ, Aplin JD, Abrahams VM (2012). Immune cell activation by trophoblast-derived microvesicles is mediated by syncytin 1. Immunology.

[28] Mincheva-Nilsson L, Baranov V, Nagaeva O, Dehlin E (2016). Isolation and characterization of exosomes from cultures of tissue explants and cell lines. Curr Protoc Immunol.

[29] Macchi V, Stocco E, Stecco C, Belluzzi E, Favero M, Porzionato A, De Caro R (2018). The infrapatellar fat pad and the synovial membrane: an anatomo-functional unit. J Anat.

[30] Eymard F, Pigenet A, Citadelle D, Tordjman J, Foucher L, Rose C, Flouzat Lachaniette CH, Rouault C, Clement K, Berenbaum F (2017). Knee and hip intra-articular adipose tissues (IAATs) compared with autologous subcutaneous adipose tissue: a specific phenotype for a central player in osteoarthritis. Ann Rheum Dis.

